# Antimicrobial and Antibiofilm Properties of Graphene Oxide on *Enterococcus faecalis*

**DOI:** 10.3390/antibiotics9100692

**Published:** 2020-10-13

**Authors:** Cecilia Martini, Francesca Longo, Raffaella Castagnola, Luca Marigo, Nicola Maria Grande, Massimo Cordaro, Margherita Cacaci, Massimiliano Papi, Valentina Palmieri, Francesca Bugli, Maurizio Sanguinetti

**Affiliations:** 1 Dipartimento di Scienze Biotecnologiche di Base, Cliniche Intensivologiche e Perioperatorie, Università Cattolica del Sacro Cuore, 20123 Rome, Italy; cecilia.martini01@icatt.it (C.M.); margherita.cacaci01@icatt.it (M.C.); francesca.bugli@unicatt.it (F.B.); maurizio.sanguinetti@unicatt.it (M.S.); 2 UOC Odontoiatria Generale e Ortodonzia, Dipartimento Scienze dell’Invecchiamento, Neurologiche, Ortopediche e della Testa Collo, Fondazione Policlinico Universitario “A. Gemelli” IRCCS, 00168 Rome, Italy; frankie1370705@hotmail.it (F.L.); luca.marigo@unicatt.it (L.M.); nicolamaria.grande@unicatt.it (N.M.G.); massimo.cordaro@unicatt.it (M.C.); 3 Istituto di Odontoiatria e Chirurgia Maxillo-Facciale, Università Cattolica del Sacro Cuore, 00168 Rome, Italy; 4 Dipartimento di Scienze di Laboratorio e Infettivologiche, Fondazione Policlinico Universitario “A. Gemelli” IRCCS, 00168 Rome, Italy; 5 Fondazione Policlinico Universitario “A. Gemelli” IRCCS, 00168 Rome, Italy; massimiliano.papi@unicatt.it (M.P.); valentina.palmieri@unicatt.it (V.P.); 6 Dipartimento di Neuroscienze, Università Cattolica del Sacro Cuore, 00168 Rome, Italy; 7 Istituto dei Sistemi Complessi (ISC), Consiglio Nazionale delle Ricerche (CNR), 00185 Rome, Italy

**Keywords:** antimicrobial properties, biofilm, graphene oxide, *Enterococcus faecalis*, radicular dentin

## Abstract

The aim of this study was to evaluate the antibacterial properties of graphene oxide (GO) against *Enterococcus faecalis* in vitro conditions and when used to coat dentin surface to prevent *E. faecalis* adhesion. The ATCC strain of *E. faecalis* 29212 has been used to perform a viability test. The pellet was suspended in ultrapure water, NaCl, PBS buffer, CaCl_2_ and MgCl_2_, Luria−Bertani broth solutions. The viability was evaluated by the colony forming unit counting method. Atomic force microscopy images and the measure of surface zeta potential variation were analyzed. Dentin discs were covered with a film of GO (*n* = 15) or were not treated (*n* = 15). Bacterial suspension was added to each sample of dentine discs and microbial counts were calculated. Statistically significant differences between two groups were assessed by a two-tailed unpaired *t*-test. Bacteria cell morphology was investigated with scanning electron microscopy. The highest growth inhibition was obtained in ddH_2_O and CaCl_2_ solution while, in PBS and NaCl, GO had poor antibacterial efficacy with a growth enhancing effect in the latter. GO on dentin discs demonstrated high antibacterial activity. GO film has demonstrated acceptable adhesion properties to root dentin and a role in the inhibition of bacterial film proliferation and biofilm formation.

## 1. Introduction

The main goal in the management of endodontic infections is to eliminate the microbial biofilm from the root canal system and to provide favorable root canal sealing. An adequate and immediate restoration after endodontic treatment is crucial to prevent reinfection of the root canal space [1]. Moreover, severe diseases could be probably associated to endodontic infections [2]. Although mechanical instrumentation, irrigation and medications have a critical role in microorganisms’ reduction in the root canal, they are not completely successful in eliminating strongly attached microorganisms from the root system. Hence, research is focused on the development of new coating material that can prevent the adhesion and the biofilm formation of microorganisms on medical devices [3,4,5]. Compared to planktonic, the biofilm mode allows bacteria to survive long period on the surfaces with low nutrient need as they are in a starvation state [6]. The cells in biofilm are more resistant to antimicrobial therapies and to the host immune response making these infections hard to be treated, as antibiotics are usually active against planktonic cells that are actively reproducing. In recent years, the use of metal or carbon nanoparticles has become an interesting approach to prevent microbial adhesion: they appear to be safe, not expensive allowing to overcome the issue of the antibiotic resistance [7]. Among the carbon nanomaterial, graphene presents several physical properties: stretch ability, electrical conductivity, huge surface area and high thermal conductivity [8,9]. Since the synthesis of graphene, many derivatives have been studied, such as graphene oxide (GO) and reduced GO (rGO). Particularly, GO, a precursor of large-scale graphene synthesis, has attracted attention for its multi-targeting killing strategy, simple production and low cost. Three main antimicrobial activities [10] have been reported:

(i) GO sheets sharp edges can physically interfere with a microorganism by cutting bacteria membranes (nano-knife or nano-blade effect) [11];
(ii) GO can induce oxidative stress [12,13];
(iii) GO can wrap and isolate microorganisms from the environment so that they cannot find nutrition, stopping proliferation [14].

There are numerous potential applications of GO in biomedicine, as biological and molecular imaging, drug/gene delivery, cancer therapy, tissue scaffold and as an antibacterial agent [15,16]. It seems to be an interesting material also for dental use and, in particular, in the endodontic/restorative fields [17,18]. The presence of residual microorganisms in the root canal system results in treatment failure, post-treatment disease, unpredictable damage and an adverse outcome in regenerative endodontic procedures. *Enterococcus faecalis* is a Gram-positive commensal bacterium, commonly associated to persistent endodontic infections. The expression of virulence determinants of *E. faecalis*, such as genes involved in bacterial adherence (gelE and efaA), biofilm formation (esp) and biofilm development (fsrC) ultimately leads to resistance against root canal irrigants and intracanal medicaments. Therefore all this has an important role in the high rate of treatment failure among endodontic infections [19,20,21]. In this paper, GO was selected as a substrate due to its wide use and environmental safety. Antimicrobial activity and cytotoxicity of GO has been described in many studies [22,23]. Palmieri et al. [24] reported how GO antibacterial effects can be modulated by the surrounding solution in order to facilitate GO clinical applications. In this paper, GO antibacterial properties against *E. faecalis* were first analyzed in extra physiological conditions, taking into account the different external conditions for the evaluation of antimicrobial tests colony forming units (CFU) counting, then atomic force microscopy (AFM) images and the measure of surface zeta potential variation were analyzed. Based on these findings and considering the important role of *E. faecalis* in the failure of endodontic treatment we decided to evaluate potential antibacterial properties of GO when used to coat the dentin surface to prevent *E. faecalis* adhesion.

## 2. Results

### 2.1. Effect of Environment Surrounding on GO Activity Against E. faecalis

**Figure 1:**
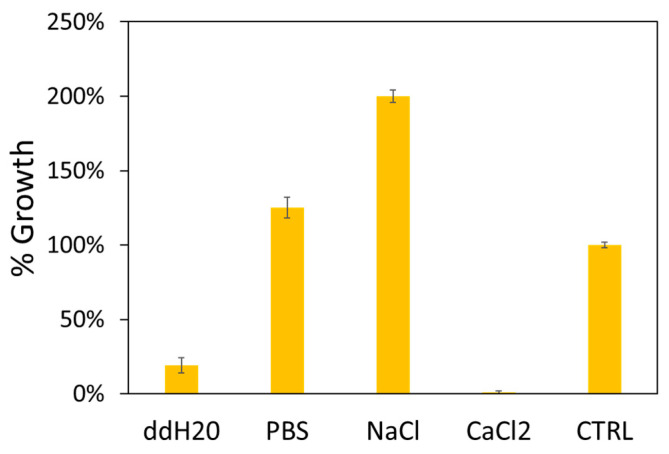
Normalized colony forming units (CFU) assay results of *Enterococcus faecalis* exposed to GO in ddH_2_O, PBS, NaCl and CaCl_2_ solutions compared to untreated samples (CTRL).

In order to evaluate the different effects of GO against *E. faecalis*, we decided to analyze the vitality of bacterial cells in contact with GO in different solutions. NaCl, PBS, CaCl_2_ and ddH_2_O were tested since it has been demonstrated that GO has different stability and, therefore, different effects on bacteria depending on the surrounding solutions and their concentration (Table 1) [24]. In Figure 1 the number of CFU normalized to control samples (bacteria growth in different solutions without GO) in the different solutions is reported as % values. The highest growth inhibition was obtained in ddH_2_O and CaCl_2_ solution. While in PBS and NaCl, as expected from prior results [25], GO has poor antibacterial efficacy with a growth enhancing effect in the latter.

### 2.2. AFM Results

**Figure 2:**
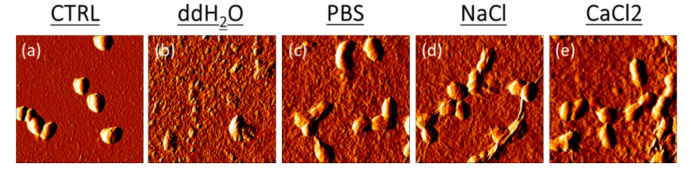
Representative AFM error images of *E. faecalis* after GO treatment in ddH_2_O, PBS, NaCl and CaCl_2_ solutions compared to untreated cells.

**Figure 3:**
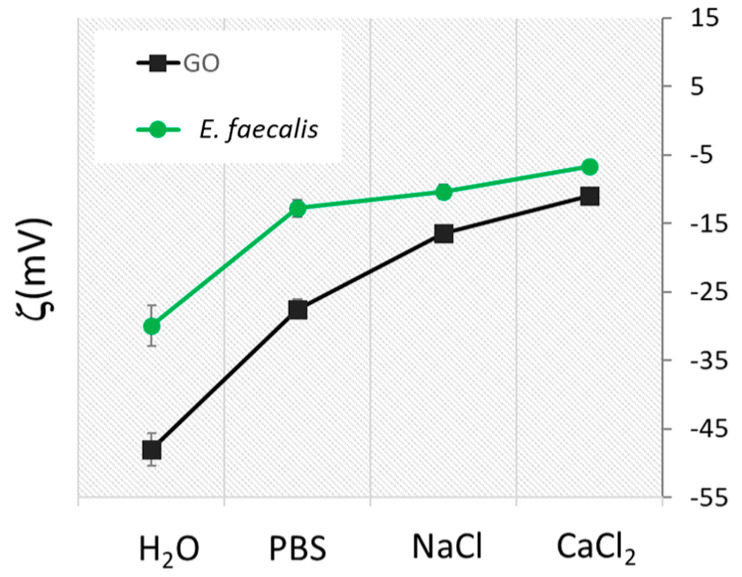
Zeta potential analysis of GO and *E. faecalis* in different solutions, GO concentration as set at 100 µg/mL.

In ddH_2_O, GO had a bacteriolytic effect resulting in the overall disruption of the cells by membrane cut/oxidation causing the leakage of intracellular material and a loss of bacterial integrity (Figure 2). In PBS, NaCl and CaCl_2_, GO had a bacteriostatic effect on the cells that did not lose integrity but in the latter, as already shown with CFU results in Figure 1, growth was inhibited due to a strong interaction with the GO surface [25]. To explain this effect, we evaluated surface charge properties of bacteria and GO in different solutions by Zeta potential measurements. The Zeta potential technique can estimate the surface charge, which can be employed for understanding the physical stability and the repulsion between particles in solution. A large positive or negative value of zeta potential indicates electrostatic repulsion [12]. In this case, in water there is a repulsive force between bacteria and GO. On the other hand, a small zeta potential value could result in particle attraction. As shown in Figure 3, starting from a negative ζ of −45 mV, the GO surface adsorbed positive ions in a solution of PBS, NaCl and CaCl_2_. Positive ions were also absorbed by the negative charged bacteria membrane. In water, GO and bacteria had repulsive interactions, favoring the impact of bacteria with GO blades and membrane cutting. In PBS and NaCl, interactions GO and bacteria flocculated with weak interactions and *E. faecalis* growth was fostered on aggregated GO scaffolds. In CaCl_2_, Zeta potential decreased and strong bridges between the GO surface and bacteria cells formed. This caused the immobilization of bacteria cells on the GO surface. A similar effect has been observed with *E. coli* and *S. aureus* [12].

### 2.3. Assessment of E. faecalis Viability on Treated and Untreated Dentine Discs by CFU Determination

**Figure 4:**
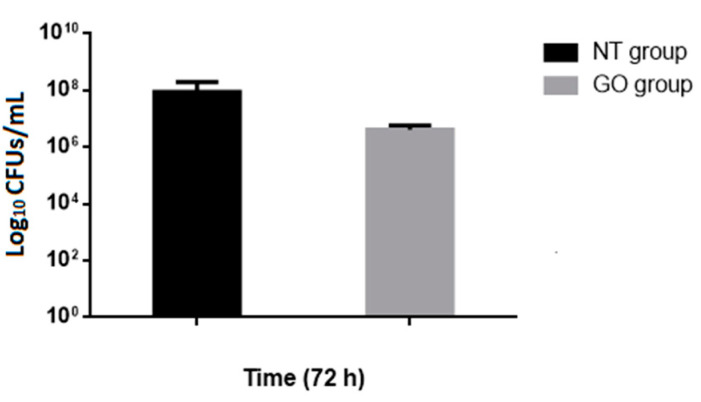
Analysis of the viability of *E. faecalis* cells on dentin surfaces uncoated (non-treated samples, NT) and coated by GO (GO group samples). Adherent bacteria were collected by sonication and plated on Enterococcus Selective Agar (ESA) to count viable cells. Data are expressed as the log10 CFUs and bars indicated standard deviation. Log10 counts were compared for statistical significance by a two-tailed unpaired *t*-test. *p* < 0.05 was considered significant. A statistically significant difference was found between treated and untreated groups *p* = 0.022).

In this study, we focused our attention on the use of GO-coated surfaces to prevent bacterial biofilm formation. Based on the previous results, antiadhesion and antibiofilm properties of GO were evaluated in GO-coated and GO-uncoated dentine discs by the colony forming count method (Figure 4). After 72 h of incubation, GO demonstrated high antibacterial activity (48 h CFU determination was not shown); Figure 4 showed that, after 72 h of incubation in the presence of GO, the number of bacteria decreased dramatically indicating the role of GO nanomaterial in the inhibition of bacterial adhesion, a crucial step toward the formation of the biofilm. To support these results we also performed the crystal violet assay for determining the entire biomass of the biofilm. However, in our practical experience, we had to take care of the experimental artifacts, formed from the non-specific interaction of the crystal violet with root dentine, to avoid unintended errors in our observations/results (data not shown).

### 2.4. Scanning Electron Microscopy Confirms the Prepared ex vivo Biofilms Model

**Figure 5:**
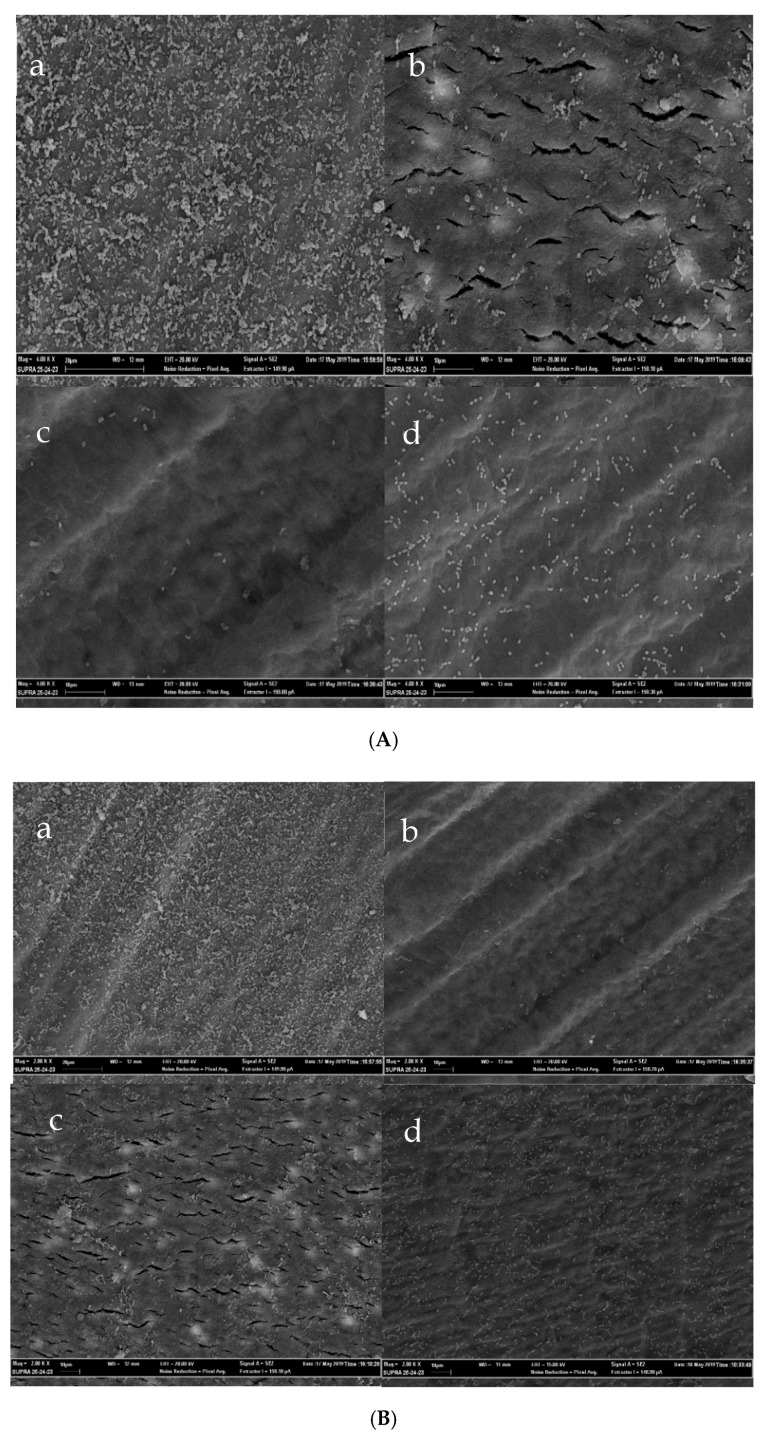
(**A**): SEM images of *E. faecalis* cells adhered to the dentin surface; (**a**) control samples at 4000× and (**b**–**d**) GO coated dentin disks at 4000×. (**B**): SEM images of *E. faecalis* cells adhered to the dentin surface; (**a**) control samples at 2000× and (**b**–**d**) GO coated dentin disks at 2000×.

To further confirm the antibiofilm properties of GO, bacterial adhesion and biofilm formation on the two groups were analyzed using scanning electron microscopy (SEM). Images from the untreated and treated dentine discs are shown in Figure 5. As shown in the Figure 5, an organization in cellular aggregates (microcolonies) was formed by *E. faecalis* in the non-functionalized control samples, on the contrary, in the surface coated with GO, bacterial cells were spread around without any aggregative structure. The ECM is not so evident in the biofilm formed, probably due to insufficient magnification. Overall, the different bacterial adhesion to GO-treated and untreated surfaces was extremely convincing and the antibiofilm effect exerted by the GO appeared substantial.

## 3. Discussion

In this study the in vitro antimicrobial properties of GO against *E. faecalis* and its possible use as a coating on the root dentin was evaluated.

*E. faecalis* was chosen as an infectious agent because it is commonly associated with failure of endodontic treatments and it is mainly isolated from the patient showing persistent apical periodontitis [26].

Its prevalence in such infections ranges from 24% to 77%, which can be explained by various survival and virulence factors belonging to *E. faecalis*, including its ability to compete with other microorganisms, invade dentinal tubules and resist nutritional deprivation. The increase of the apical preparation sizes and inclusion of 2% chlorhexidine in combination with sodium hypochlorite are currently the most effective methods to eliminate *E. faecalis* within the root canal systems [27].

GO represents a carbon nanomaterial that shows antibacterial activity either alone or in combination with other substances [10]. Few studies focused on the possibility to use GO as a coating to prevent bacterial adhesion [28,29,30] showing a reduction of adhesion and viability in the GO-functionalized samples compared to the control. Additionally, the antibiofilm activity has been investigated, Farid et al. [31] in 2018 showing either a mechanical disruption and intracellular induction of reactive-oxygen species (ROS) of *Escherichia coli* and *Staphylococcus aureus* cells exposed to the GO coated PDVF membrane compared to the control. Authors also measured the thickness of the biofilm, showing a decreased thickness in the treated sample that demonstrated a role in the inhibition of the biofilm formation. Overall graphene’s antimicrobial properties against *E. faecalis* have been investigated in the literature. For example, Pulingam et al. were the first to report a difference in the activity of GO towards Gram-positive and Gram-negative bacteria. They have shown that GO antibacterial activity towards *S. aureus*, *E. faecalis*, *E. coli* and *Pseudomonas aeruginosa* is concentration- and time-dependent. The GO antibacterial mechanism differed between Gram-positive and Gram-negative bacteria, where the majority of bacterial inactivation of Gram-positive bacteria occurs through the bacterial wrapping mechanism. On the other hand, inactivation of Gram-negative bacteria mainly occurs through physical contact, which leads to membrane damage [32]. In this study, we tested the properties of the GO in various in vitro conditions against *E. faecalis*, confirming the importance of the environment in which it acts. After obtaining these encouraging results, we evaluated the GO’s ability to inhibit the adhesion of the bacterium on a biological surface such as GO-functionalized dentin. We decided to assess the experimental condition for the biofilm formation, up to 72 h, time that seems enough to have a high percentage of infiltration in dental tubules [33,34]. We have shown how the biofilm formation in the presence of GO is impaired with respect to the GO untreated samples, indicating the role of GO nanomaterials in the inhibition of bacterial film proliferation.

The presence of the GO surface may not provide sufficiently attractive surface properties for anchoring of bacteria thereby interfering with the cell−surface interactions and inhibiting bacterial attachment, which is the primary requirement for the establishment of a biofilm. The GO-uncoated control surface showed an organization in cellular aggregates (microcolonies) of *E. faecalis*. In this case, the dentine surface possesses a particular composition and structure, which is conducive to the formation of biofilm by facilitating the anchoring mechanism. On the contrary, the GO-coated surface mediates the growth locally creating a partially covered surface, however further growth and transition from planktonic growth to the biofilm are inhibited.

The use of dentin discs is a limitation of this study and it would be desirable to test GO on extracted teeth in order to better reproduce in vitro a clinical scenario. Further studies about the interaction between GO and radicular dentin are required, in order to better understand this aspect and enhance the adhesion between the two surfaces. It has been reported that oxidative stress has an important role in the antibacterial mechanisms of GO and it is caused by the presence of the sheet’s defects that enhance GO reactivity to biomolecules [14,35]. Moreover, further studies are required in order to clarify the ideal dimension, concentration and shape of GO sheets that better express their antimicrobial effect against different persistent bacteria and the biocompatibility of GO and to state whether it is suitable for dental clinical practice and it could be used in the endodontic field. However the effect of GO on human oral tissues remains unknown yet and it must be explored prior to any dental clinical practice.

## 4. Materials and Methods

### 4.1. GO, Bacterial Strains and Growth Media

GO stock solutions have been prepared from a stock solution at 4 mg/mL (Graphenea) and stored at room temperature protected by light. GO dilutions have been always prepared using ultrapure deionized water. GO (Graphenea, Cambridge, MA, USA) solutions were prepared at different concentrations and sonicated for 30 min with a probe-sonicator at 50 W (VC50 Sonics and Materials UK) to reduce sample polydispersity. The ATCC strain of *Enterococcus faecalis* 29,212 was used in this study. The strain was grown in brain heart infusion broth (BHI, Sigma) supplemented with 0.25% glucose and 1% fetal calf serum (FBS, Sigma) at 37 °C overnight.

### 4.2. In Vitro GO Activity Against E. faecalis

*E. faecalis* (ATCC strain 29212) has been used to perform a viability test. Bacteria were grown in LB medium at 37 °C overnight, and then bacteria cells were harvested via centrifugation (4000 rpm for 10 min). Bacteria cells were washed three times with deionized water to remove residual macromolecules and other growth medium constituents. The pellet was then suspended in ultrapure water, NaCl solution (0.9%), PBS buffer, CaCl_2_ (10 mM) and MgCl_2_ (10 mM), Luria−Bertani (LB) broth solutions. Hereafter, we will refer to these buffers by using ddH_2_O, NaCl, PBS, CaCl_2_, MgCl_2_ and LB. Bacterial cell suspension was diluted in the specific buffers or solutions to obtain cell samples having a McFarland turbidity of 0.5 corresponding to 10^8^ CFU/mL. Of the cells 180 μL in different buffers was incubated with 20 μL of fresh GO suspensions at a concentration ranging from 3 to 200 μg/mL, at room temperature for 4 h without shaking or for 24 h in LB broth. Growth in LB broth was followed by measuring optical density with Cytation 3 Cell Imaging Multi-Mode Reader. The viability was evaluated by the colony counting method. Briefly, a series of 10-fold cell dilutions (100 μL each) were spread onto LB plates and allowed to grow overnight (12 h) at 37 °C. Colonies were counted and compared to those on control samples (untreated samples) to calculate changes in the cell growth inhibition.

### 4.3. Zeta Potential

To characterize repulsion between bacteria and GO, samples were characterized with dynamic light scattering with Zetasizer Nano ZS (Malvern, Herrenberg, Germany) equipped with a 633 nm He−Ne laser and operating at an angle of 173°. The zeta potential was calculated from the electrophoretic mobility by means of the Henry correction to Smoluchowski’s equation. Data analysis was performed by Malvern Zetasizer software.

### 4.4. AFM

AFM has been used to observe at high resolution the effects of GO on bacteria morphology without altering the samples by complex dehydration and metallization. For AFM measurements, samples were prepared as explained elsewhere [36]. Briefly, 20 μL of GO were deposited on sterile cover glass slides, air-dried and washed once with ultrapure water (37 °C) to remove salt deposits interfering with the measurements. After sample preparation, measurements were immediately performed with a NanoWizard II AFM (JPK Instruments AG, Berlin, Germany). The images were acquired using silicon cantilevers with high aspect-ratio conical silicon tips (CSC37 Mikro-Masch, Tallinn, Estonia) characterized by an end radius of about 10 nm and a half conical angle of 20°. Cantilevers with a nominal spring constant of 0.4 *n*/m were accurately calibrated.

### 4.5. Dentin Discs Sample Preparation

For this study, 20 single rooted maxillary teeth were collected. Teeth were extracted for orthodontic or periodontal reasons and the following inclusion criteria were applied:

(1) Fully-formed roots and apex,
(2) Absence of anatomic anomalies or canal calculus (assessed with radiographs in vestibular-palatal and mesio-distal projections),
(3) Absence of previous endodontic treatment,
(4) Absence of caries or fracture.

All patients signed an informed consent agreement. After extraction, calculus and tissue debris were removed from teeth using manual curettes and all teeth were stored in saline solution (NaCl 0.9%) in order to prevent dehydration. All treatments were performed by the same clinician. The traditional access approach was performed using diamond burs mounted on turbine and tungsten carbides mounted on the handpiece contra-angle with water-cooling. An ISO #10 and #15 K-file (Dentsply Maillefer, Ballaigues; Switzerland) were used to determine the working length. All root canals were instrumented with ProTaper Next (Denstply Maillefer) up to a final size of 40.06. Crown-down manner was performed according to the manufacturer’s instructions. During cleaning and shaping, 5.25% sodium hypochlorite (Niclor 5, OgnaLab S.r.l, Muggiò, Italy) was used after each instrument size. At the end of the shaping phase canals were filled with 17% EDTA solution (Stomygen, Coswell Grop, Funo, Italy) for 30 s in order to remove the smear layer and debris. In the end, a final rinse was performed with saline solution (NaCl 0.9%) and canals were wiped using air and absorbent points. The teeth were sliced using a 0.2 mm low-speed diamond disk under water-cooling. The cuts were performed from the cementoenamel junction to the apex and 3 mm thick discs were obtained from the root of each sample. Every disc was evaluated using a caliper in order to obtain standardize samples. Every disc was attached to circular cover slips (ø13 mm) and sterilized using PLASMA sterilization (Steris V-PRO^®^ maX Low Temperature Sterilization System Group, Bordeaux, France).

After sample preparations, dentin discs were then randomly allocated in two experimental groups. The GO group (*n* = 15) was formed by dentin discs covered with a film of GO. A GO liquid suspension was used (^®^Graphenea—graphene oxide water dispersion, 0.4 wt % concentration, San Sebastián Spain). Firstly, the suspension was diluted with deionized water at a concentration of 1 mg/mL and then sonicated in a water bath sonicated for 30 min in order to obtain a homogeneous and stable suspension. On the surface of every dentin disc 100 μL of GO suspension was added and the samples were left dried for 24 h at room temperature (drop casting method) [37]. After 24 h the solvent was completely evaporated and a thin film of GO was obtained on the surface of every sample. No treatments were performed for the control samples (*n* = 15).

### 4.6. Biofilm Formation Assay

Biofilm formation by *E. faecalis* isolates was performed according to the reference method with modifications [38]. Briefly, *E. faecalis* isolates were cultivated in BHI (BHI, Sigma) supplemented with 0.25% glucose and 1% fetal calf serum (FBS, Sigma) at 37 °C in ambient air for 10–12 h without shaking. Overnight cultures were then diluted to reach 0.5 McFarland (corresponding to 1.5 × 10^8^ CFU/mL) in 500 µL of BHI supplemented with 0.25% glucose and 1% fetal calf serum.

Bacterial suspension, prepared as described before, was added to each sample of dentine discs that were allocated in 24-Wells Microtiter Plate (Costar3527, Corning). Samples were incubated for 72 h at 37 °C in order to allow the formation of biofilm. Dentin discs were gently washed to remove not adhered bacteria. After washing the samples, the biofilm was detached from the surfaces by bath sonication. Bacterial cell suspensions were then serial diluted and spread on ESA plates (ESA, Sigma, St. Louis, MO, USA) and were incubated at 37 °C overnight. After 24 h, microbial counts were expressed as CFU per mL.

Results were statistically analyzed in GraphPad Prism (version 5.0, GraphPad Software Inc., San Diego, CA, USA). Statistically significant differences between two groups were assessed by a two-tailed unpaired *t*-test (*p*-value < 0.05).

### 4.7. SEM Analysis

SEM (SEM Supra 25—Zeiss, Germany) was performed to analyze bacteria cells morphology and distribution over large dentine discs. The SEM used had a resolution of 1.7 nm at 15 KV and 3.5 nm at 5 KV, which allows magnification from 12 to 500,000×. Samples were allowed to form biofilm on treated and untreated surfaces as described before. Discs were prepared for SEM evaluation by fixation with 2.5% glutaraldehyde in phosphate buffer and post-fixation with 1.33% osmium tetroxide. Fixation was followed by dehydration of samples and finally they were mounted on stubs and sputter-coated with gold (Balzers Union—SCD 040) and imaged by SEM with a magnification of 2000×, 4000× and 6000×.

## 5. Conclusions

Within the limits of an in vitro study the antimicrobial and antibiofilm properties of GO against *E. faecalis* were confirmed. GO film demonstrated a role in the inhibition of bacterial film proliferation and it showed acceptable adhesion properties to root dentin, without causing alteration of its structure. GO is a promising material and its potential use in endodontics should be investigated.

## Figures and Tables

**Table 1:** Mean CFU counts after exposure to GO in ddH_2_O, PBS, NaCl and CaCl_2_ solutions.

	GO (ddH_2_O)	GO (PBS)	GO (NaCl)	GO (CaCl_2_)	CTRL
*E. faecalis* (CFU × 10^5^)	26	171	273	1	173
